# Novel method for screening functional antibody with comprehensive analysis of its immunoliposome

**DOI:** 10.1038/s41598-021-84043-w

**Published:** 2021-02-25

**Authors:** Shusei Hamamichi, Takeshi Fukuhara, Izumi O. Umeda, Hirofumi Fujii, Nobutaka Hattori

**Affiliations:** 1 Research Institute for Diseases of Old Age, Juntendo University School of Medicine, Tokyo, 113-8421 Japan; 2 Department of Neurology, Juntendo University Graduate School of Medicine, 2-1-1 Hongo, Bunkyo-ku, Tokyo, 113-8421 Japan; 3 Department of Research for Parkinson’s Disease, Juntendo University Graduate School of Medicine, Tokyo, 113-8421 Japan; 4 Kavli Institute for the Physics and Mathematics of the Universe, The University of Tokyo, Kashiwa, Chiba 277-8583 Japan; 5 Division of Functional Imaging, Exploratory Oncology Research and Clinical Trial Center, National Cancer Center, Kashiwa, Chiba 277-8577 Japan

**Keywords:** Biomaterials, Drug delivery

## Abstract

Development of monoclonal antibody is critical for targeted drug delivery because its characteristics determine improved therapeutic efficacy and reduced side-effect. Antibody therapeutics target surface molecules; hence, internalization is desired for drug delivery. As an antibody–drug conjugate, a critical parameter is drug-to-antibody ratio wherein the quantity of drugs attached to the antibody influences the antibody structure, stability, and efficacy. Here, we established a cell-based immunotoxin screening system to facilitate the isolation of functional antibodies with internalization capacities, and discovered an anti-human CD71 monoclonal antibody. To overcome the limitation of drug-to-antibody ratio, we employed the encapsulation capacity of liposome, and developed anti-CD71 antibody-conjugated liposome that demonstrated antigen–antibody dependent cellular uptake when its synthesis was optimized. Furthermore, anti-CD71 antibody-conjugated liposome encapsulating doxorubicin demonstrated antigen–antibody dependent cytotoxicity. In summary, this study demonstrates the powerful pipeline to discover novel functional antibodies, and the optimal method to synthesize immunoliposomes. This versatile technology offers a rapid and direct approach to generate antibodies suitable for drug delivery modalities.

## Introduction

In recent years, antibody–drug conjugates (ADCs) in which monoclonal antibodies are attached to cytotoxic payloads by non-cleavable or cleavable linkers have emerged as highly potent pharmaceutical modalities because of target specificity and target-binding affinity that collectively contribute to targeted delivery of the drugs as well as reduced side effects^1–3^. Among these components, monoclonal antibodies are critical because therapeutic properties of ADCs are partially dependent on the characteristics of their antigens. The efficacies of ADCs are primarily dependent on the expression patterns of the targeted antigens; however, recent reports revealed that successful ADCs possess internalization properties that will facilitate them to be transported into the cells and enhance their pharmacological effects^4,5^. Therefore, a proper screening process to identify suitable monoclonal antibodies is essential for development of ADCs.

Traditionally, common assays to screen for monoclonal antibodies include enzyme-linked immunosorbent assay, flow cytometry, and immunoblotting analyses. While these procedures may assist in characterizing target specificity and target-binding affinity, they are not suitable to determine internalization properties of the antibody. To this end, we previously reported a cell-based immunotoxin screening system to facilitate the isolation of functional antibodies with internalization capacities^6^. Functional antibodies were selected from the hybridoma library through formation of immunotoxins via Fc-mediated coupling of antibodies with engineered diphtheria toxin DT3C, and screening for conditional lethality of the target cells if the immunotoxins were internalized. This rapid and straightforward screening strategy possesses the following advantages: 1) selection of potent antibodies that effectively bind to the cell-surface epitopes, and 2) selection of the antibodies that are efficiently internalized into the cells. Collectively, our approach allows direct discovery of potent ADC-compatible antibodies.

In this study, we generated anti-U87 (human glioblastoma) hybridoma library through conventional immunization and cell fusion. Next, secreted antibodies found in the hybridoma supernatants were pre-formed with DT3C, and these immunotoxins were subjected for screening of potent antibodies through administration to U87 cells. We herein report the successful isolation of a functional monoclonal antibody targeting the surface molecule of U87 cells with internalization capacity. In the form of ADCs, a critical parameter as a drug delivery vehicle is drug-to-antibody ratio (DAR). High DAR may influence antibody structure and stability whereas low DAR could reduce efficacy; hence in most ADCs, their DAR values are restricted at the controlled level^7–9^. To maximize drug load while minimizing structural changes and instability, we conjugated the functional antibody to liposome to generate immunoliposome. Through optimization of liposomal composition, we observed enhanced cellular uptake of antibody-conjugated liposome in an antigen–antibody binding dependent manner when compared with control mouse IgG (mIgG)-conjugated liposome. Furthermore, we observed enhanced cytotoxicity of the immunoliposome encapsulating anti-cancer agent doxorubicin in the antigen–antibody binding dependent manner. Taken together, our screening strategy allowed rapid identification of functional antibody with potential application, including immunoliposome, as a novel modality of drug delivery systems.

## Results

### Immunotoxin screening of anti-U87 hybridoma library identified 214D8 clones

**Figure 1:**
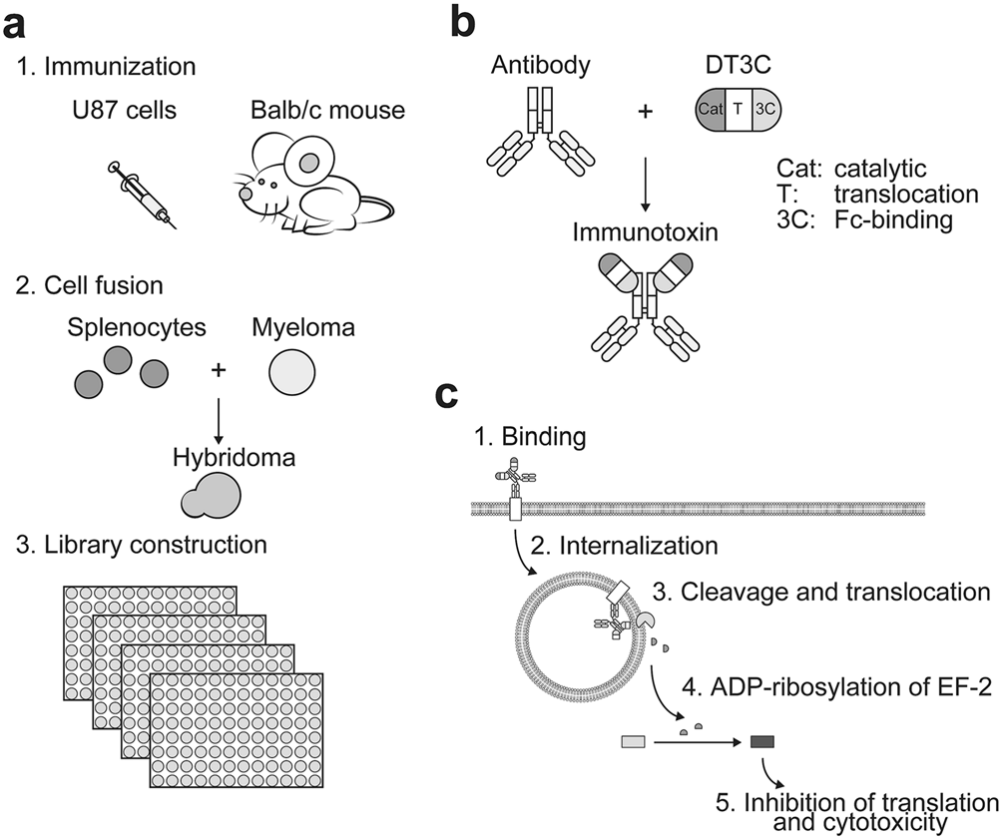
Screening pipeline. **(a)** Generation of anti-U87 hybridoma library. A hybridoma library was generated through: (1) immunization of BALB/c mice, (2) cell-fusion of splenocytes and myeloma cells, and (3) library construction of hybridomas by HAT selection. **(b)** DT3C mediated immunotoxin formation. Hybridoma supernatants containing antibody were pre-incubated with engineered toxin DT3C to form antibody:DT3C immunotoxin via Fc-binding domain (3C). **(c)** Mechanism of antibody:DT3C-induced cytotoxicity. The potent antibody:DT3C immunocomplex binds to the antigens expressed on the cell surface. The immunotoxin is then internalized wherein translocated terminus of DT3C is cleaved by the cellular furin protease, and catalytic domain of DT3C (Cat) is released into the cytoplasm. Subsequently, the catalytic domain ADP-ribosylates elongation factor (EF)-2, which leads to cytotoxicity via inhibition of the protein translation machinery.

A hybridoma library was generated through immunization of BALB/c mice with U87 cells, fusion of immunized splenocytes and P3U1 myeloma cells, and selective growth of hybridomas in the 96-well plates containing culture media with hypoxanthine-aminopterin-thymidine (HAT) (Fig. 1a). In total, 312 out of 384 wells (81%) contained at least one colony of hybridoma cells (data not shown). Supernatants from the hybridoma library were pre-incubated with engineered toxin DT3C to form immunotoxins (Fig. 1b). DT3C is a recombinant protein that consists of diphtheria toxin (DT) without the receptor-binding domain but containing the Fc-binding domains of *Streptococcus* protein G (3C)^10^. As summarized in Fig. 1c, if the antibody:DT3C immunocomplex recognizes an antigen expressed on the cell surface, then the immunotoxin is internalized wherein DT3C is cleaved by the cytosolic furin protease, and catalytic domain of DT3C is released into the cytoplasm. The catalytic domain causes ADP-ribosylation of elongation factor (EF)-2, which subsequently leads to cytotoxicity via inhibition of the protein translation machinery.

Exploiting the unique principle of DT3C immunotoxin assay, we performed primary screening to identify antibody-secreting hybridomas that were capable of inducing DT3C-dependent cytotoxicity. Visual observation of U87 morphology, as well as immunotoxin assay revealed 214A2 and 214D8 as putative hybridomas that secreted functional antibodies with DT3C-dependent cytotoxicity (Supplementary Fig. 1). These positive hybridomas were subjected to limiting dilution for cloning, and supernatants from the clones were used to perform secondary screening for confirmation. Collectively, we established 214D8 clone that produced functional antibody with capacity for DT3C-dependent cytotoxicity.

### 214D8 antibody recognized CD71/TFRC

**Figure 2:**
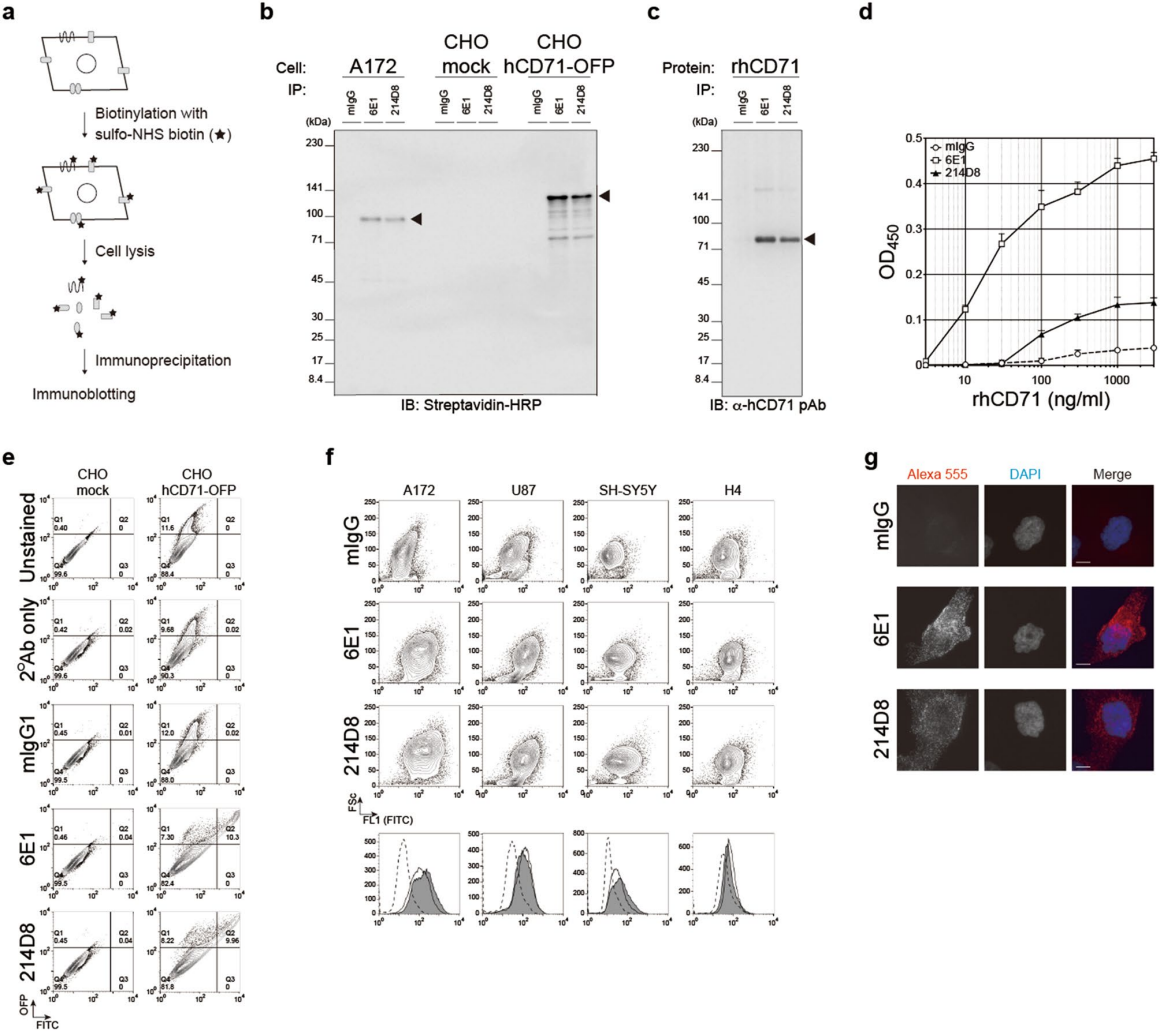
Characterization of functional antibody, 214D8. **(a)** Schematic diagram of profiling antigen. Cell surface proteins of A172, CHO mock or CHO hCD71-OFP cells were biotinylated with sulfo-NHS-biotin, and the cells were lysed to obtain biotinylated surface proteins. These proteins were subjected for immunoprecipitation and immunoblotting. **(b)** Full-length immunoblot of the biotinylated surface antigen. Following immunoprecipitation with the indicated antibodies, samples were electrophoresed, transferred, probed with streptavidin-HRP conjugate, and visualized by using chemiluminescent substrate. Putative antigen of 98-kDa band indicative of hCD71 and 125-kDa band indicative of hCD71-OFP were detected by immunoblotting. Representative results of two independent experiments. **(c)** Full-length immunoblot of purified rhCD71. Following immunoprecipitation with the indicated antibodies, samples were electrophoresed, transferred, probed with anti-hCD71 polyclonal antibody, and visualized by using chemiluminescent substrate. Putative antigen of 77-kDa band indicative of rhCD71 was detected by immunoblotting. Representative results of two independent experiments. **(d)** Detection of purified rhCD71 by sandwich ELISA. The purified rhCD71 was captured by anti-CD71 polyclonal antibody, detected by the indicated antibodies, probed with anti-mouse IgG HRP, and visualized by using TMB substrate. The mean absorbance values of quadruplicate samples at 450 nm are shown. Representative results of duplicate independent experiments. Data represent AVG ± SD. **(e)** Flow cytometry analysis of CHO cells overexpressing hCD71-OFP. The cells were stained with the indicated antibodies, and subsequently analyzed by flow cytometry. Data are visualized in scattered plots with FITC of secondary antibody and OFP of expressed hCD71-OFP protein. **(f)** Flow cytometric profiling of 214D8 antigen. Data are represented in the scattered plots of forward scatter (Fsc) and FITC (FL1) as secondary antibody of anti-CD71 antibodies including 6E1 and 214D8 as well as control mouse IgG. In histograms, data indicated FITC fluorescent intensity of control (dotted white), 6E1 (white) or 214D8 (gray) respectively. Representative data of two independent experiments. **(g)** Immunocytochemical analysis of A172 cells. The cells were stained with the indicated antibodies, followed by Alexa555 donkey anti-mouse IgG (H + L) secondary antibody, and counterstained with DAPI. 6E1 and 214D8 antibodies, but not mIgG, demonstrated membranous and cytoplasmic staining patterns. Representative results of two independent experiments. Scale bar = 10 µm. Magnification = 40x.

To identify a putative antigen of 214D8 antibody (subclass: IgG_1_ kappa), cell surface proteins were biotinylated with sulfo-NHS-biotin prior to immunoprecipitation (Fig. 2a). Glioblastoma cell line A172 was treated with sulfo-NHS-biotin, lysed with 1% NP40, and the putative antigen was subsequently immunoprecipitated by using 214D8. A 98-kDa band was detected by probing with streptavidin-HRP (Fig. 2b). We noticed similarities in the detected band patterns between 214D8 and 6E1 antibodies, a previously published antibody against human CD71 (hCD71)/TFRC^10^.

To determine if CD71 was an antigen of 214D8 antibody, purified recombinant hCD71 (rhCD71; 77.4 kDa) was immunoprecipitated by using 214D8 and 6E1 antibodies, and a 77 kDa band was detected by probing with anti-hCD71 polyclonal antibody (Fig. 2c). Additionally, sandwich ELISA also revealed immunoreactive binding between the rhCD71 and these antibodies (Fig. 2d). Human CD71-OFP expression vector was transiently transfected into the CHO cells, and these transfected cells were assessed by flow cytometry. As expected, while 214D8 and 6E1 antibodies did not react with CHO mock cells, both antibodies demonstrated reactivities against CHO hCD71-OFP cells wherein the number of OFP^+^ FITC^+^ double positive subpopulation reached 9.96% and 10.3%, respectively [Fig. 2e; quadrant 2 (Q2)]. Additionally, we performed immunoprecipitation/immunoblotting by using the cell lysates prepared from the transiently transfected CHO cells, and determined that, while 214D8 and 6E1 did not immunoprecipitate any detectable proteins in the CHO mock lysate, both antibodies immunoprecipitated a 125 kDa protein from the CHO hCD71-OFP lysate, indicative of hCD71-OFP (Fig. 2b). Based on the experimental results above, we concluded that CD71 was a genuine antigen of 214D8 and 6E1 antibody.

Next, we used flow cytometry to assess expression patterns of the antigen recognized by 214D8 antibody and found varying immunoreactive patterns against 4 cell lines, A172, U87, SH-SY5Y, and H4 (Fig. 2f). Control mIgG antibody demonstrated the following mean fluorescent intensity (MFI) values against A172 (23.7), U87 (47.6), SH-SY5Y (17.5), and H4 (53.8). Compared with the control, 214D8 antibody demonstrated strong immunoreactivities against A172 (MFI: 285), U87 (MFI: 147), and SH-SY5Y (MFI: 73.2), as well as weak immunoreactivity against H4 (MFI: 81.3). Consistent with these results, 6E1 antibody also demonstrated strong immunoreactivities against A172 (MFI: 173), U87 (MFI: 160), and SH-SY5Y (MFI: 46.3), as well as weak immunoreactivity against H4 (MFI: 89.7).

To determine the subcellular localization of CD71, A172 cells were fixed with 4% PFA, incubated with primary antibodies and subsequently with Alexa555-conjugated anti-mouse IgG secondary antibody, and counterstained with DAPI. Both 214D8 and 6E1 antibodies demonstrated similar membranous and cytoplasmic staining patterns (Fig. 2g). Taken together, these results highly suggested that CD71 was a genuine antigen of 214D8 antibody.

### Immunotoxin activity of anti-CD71 antibodies

**Figure 3:**
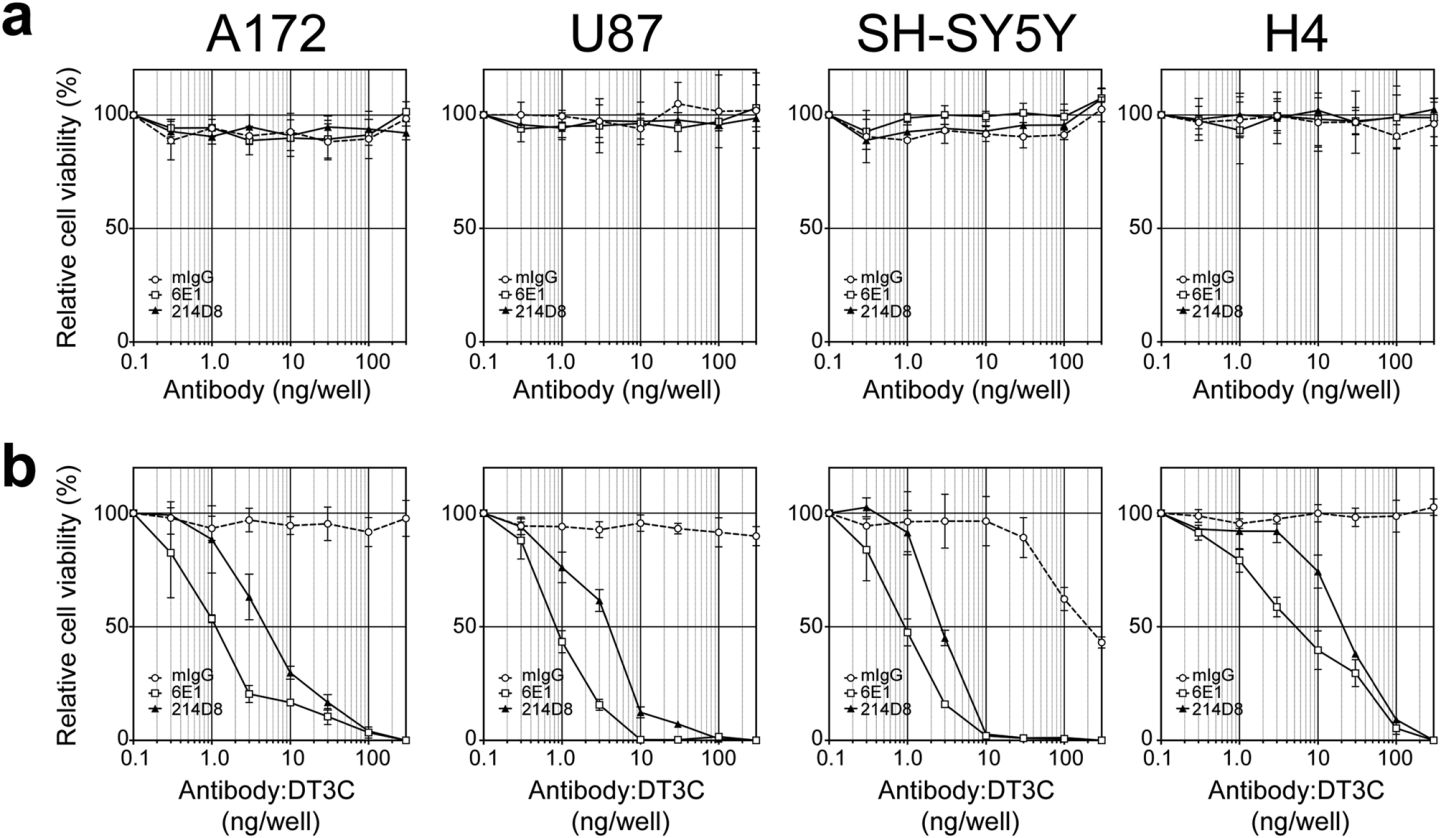
Dose-dependent activity of anti-CD71 immunotoxin. **(a)** Relative cell viability of A172, U87, SH-SY5Y and H4 cell lines after antibody treatment. Administration of antibodies did not induce cytotoxicity (n = 3 per administration). Antibody (150 kDa) at 300 ng corresponds to 13 nM. Representative results of duplicate independent experiments. Data represent AVG ± SD. **(b)** Relative cell viability of A172, U87, SH-SY5Y and H4 cell lines after antibody:DT3C treatment. Through formation of immunotoxins, both 214D8:DT3C and 6E1:DT3C induced cytotoxicity in all four cells lines tested (n = 3 per administration). Assuming that 2 DT3Cs (75 kDa each) bind to 1 antibody, antibody:DT3C at 300 ng corresponds to 13 nM. Representative results of duplicate independent experiments. Data represent AVG ± SD.

To assess cellular cytotoxicity of newly established 214D8 antibody, we treated cell lines with mIgG, 6E1, and 214D8 antibodies alone or the corresponding antibody:DT3C immunotoxins. Cytotoxicity was evaluated after incubation of the analytes for 3 days by WST-1 assay for measuring cellular viability. As shown in Fig. 3a and Supplementary Fig. 2a, administration of antibody alone did not induce cytotoxicity. On the contrary, through formation of immunotoxins, both 214D8:DT3C and 6E1:DT3C induced cytotoxicity in all four cells lines tested (Fig. 3b; Supplementary Fig. 2b). LogEC50s (ng/well) of 214D8:DT3C were 6.86 (A172), 4.27 (U87), 2.86 (SH-SY5Y), and 24.65 (H4) while superior values were observed with 6E1:DT3C at 1.51 (A172), 0.93 (U87), 0.97 (SH-SY5Y), and 7.91 (H4). These results indicated that anti-CD71 antibodies presented DT3C-dependent cytotoxicity, and that 214D8 antibody is comparable to 6E1 in immunotoxin activity.

### Anti-CD71 immunoliposome demonstrated immunoreactivity against A172 cells

**Figure 4:**
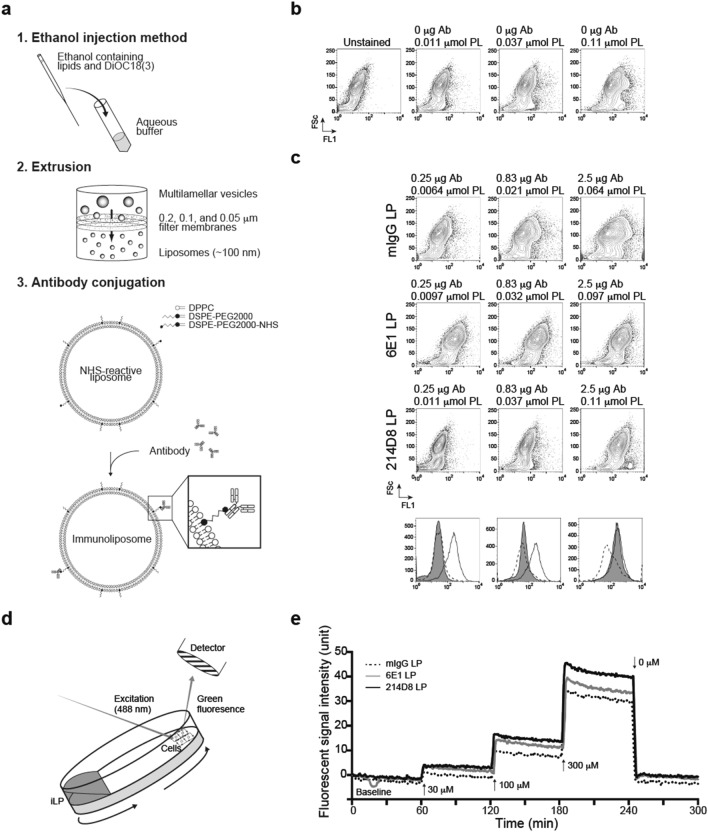
Antigen-specific binding of immunoliposomes. **(a)** Generation of immunoliposomes. Immunoliposome was generated through a three-step process: (1) the ethanol injection method, (2) extrusion through 0.05 µm, 0.1 µm, and 0.2 µm polycarbonate membrane filters, and (3) conjugation with antibody through crosslinking reaction with *N*-hydroxysuccinimide (NHS) ester. **(b)** Lack of immunoreactivity with liposome (LP) without antibody conjugation. At the phospholipid (PL) concentration equivalent of containing 0.25 µg, 0.83 µg, 2.5 µg antibody (Ab), liposome without antibody conjugation did not demonstrate reactivity against A172 cells. Representative results of duplicate independent experiments. **(c)** Immunoreactivity with immunoliposomes. A172 cells were tested by staining with control mIgG-conjugated (dotted white), 6E1-conjugated (white) or 214D8-conjugated liposomes (gray). Data of FITC fluorescent intensity (FL1) were indicated as histograms. Representative results of duplicate independent experiments. **(d)** Schematic diagram of measuring K_D_ values of immunoliposomes. LigandTracer allows real-time quantification of fluorescently labeled immunoliposome binding to immobilized cells. **(e)** Representative diagram of K_D_ measurement. Baseline, association phase, and dissociation phase through addition or subtraction of immunoliposomes were measured. Representative results of triplicate independent experiments.

Given the limited quantity of cytotoxic payloads that can be attached to monoclonal antibody, we then extended our studies by exploring application of functional antibodies through conjugation with liposome. Immunoliposome was generated by the ethanol injection method whereby lipids and DiOC_18_(3) were dissolved in ethanol and rapidly injected into aqueous buffer, extruded through 0.2 µm, 0.1 µm, and 0.05 µm polycarbonate membrane filters to produce approximately 100 nm liposome, and immediately conjugated with antibody through crosslinking reaction with N-hydroxysuccinimide (NHS) ester (Fig. 4a). Optimized procedure resulted in utilizing NHS ester and full immunoglobulin as key reaction components, leading to multiple advantages including prompt reaction as well as preserved immunoreactivity. Physical properties of the immunoliposomes are summarized in Table 1. When compared with liposome without antibody conjugation, our mIgG-, 6E1-, and 214D8-conjugated liposomes displayed slightly larger size and less negative zeta potential, but when compared amongst 3 antibody-conjugated immunoliposomes, physiological properties did not differ significantly. Importantly, antibody conjugation percentages were highly controlled: 76% for mIgG-conjugated liposome, 69% for 6E1-conjugated liposome, and 60% for 214D8-conjugated liposome.

To further confirm immunoreactivity of our immunoliposomes, A172 cells were subjected to flow cytometric analysis by using liposome without antibody conjugation at the phospholipid (PL) concentration equivalent of containing 0.25 µg, 0.83 µg, 2.5 µg antibody (Fig. 4b). These results indicated that liposome without antibody conjugation did not demonstrate reactivity. In cases of 214D8- and 6E1-conjugated liposomes, both immunoliposomes demonstrated comparable immunoreactivities at the PL concentration equivalent of containing 2.5 µg antibody (Fig. 4c). With reduction of liposomal concentration, only 6E1-conjugated liposome maintained strong signal. Median fluorescent intensity levels of these immunoliposomes are summarized elsewhere (Supplementary Fig. 3a–c). These results indicated that both 214D8- and 6E1-conjugated liposomes were immunoreactive against A172 cells.

To determine equilibrium dissociation constant (K_D_) of 214D8- and 6E1-conjugated liposomes, we utilized LigandTracer, an instrument for real-time measurement of binding of fluorescently labeled molecules to immobilized cells (Fig. 4d). We incubated A172 cells at 37 °C overnight, and subsequently measured baseline, association phase, and dissociation phase through addition or subtraction of immunoliposomes. The LigandTracer analysis revealed K_D_ values of 214D8- and 6E1-conjugated liposomes at 58.1 nM and 61.6 nM, respectively (Fig. 4e). We attempted measuring K_D_ values of Alexa488-conjugated antibodies, but we could not quantify their binding capacities due to low signal (data now shown).

### Anti-CD71 antibody-conjugated liposome demonstrated enhanced cellular uptake

**Figure 5:**
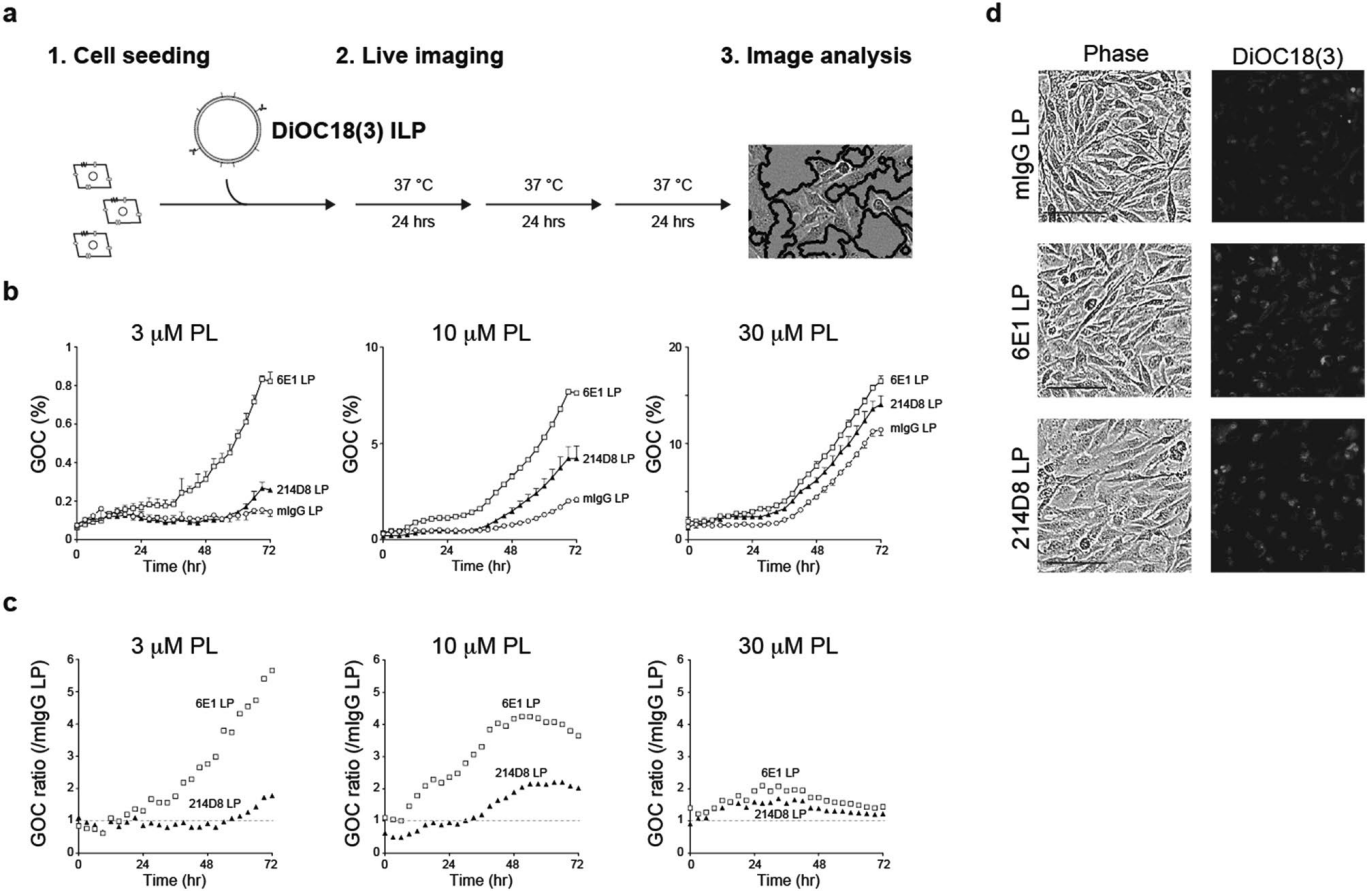
Targeted delivery of 214D8-conjugated liposome. **(a)** Schematic diagram of cellular uptake analysis. **(b)** Cellular uptake of 3 µM, 10 µM, and 30 µM antibody-conjugated liposomes (LPs). A172 cells were treated with the indicated concentrations of immunoliposomes and incubated at 37 °C for 3 days (n = 3 per administration). Concentration of immunoliposomes are presented as phospholipid concentration (µM). Antibody concentrations at the 30 µM phospholipid concentration are as follows: mIgG at 3.76 nM, 6E1 at 2.94 nM, and 214D8 at 2.79 nM. Representative results of triplicate independent experiments. Data represent AVG ± SD. **(c)** Ratio of GOC between 214D8- or 6E1-conjugated liposomes and control mIgG-conjugated liposome. At 10 µM liposomal concentration, when compared with the control mIgG-conjugated liposome, 214D8- and 6E1-conjugated liposomes exhibited 2.21- (214D8: 66 h) and 4.25-fold (6E1: 51 h) increases of the cellular uptake. Representative results of triplicate independent experiments. **(d)** Representative phase contrast and fluorescent images at 72 h after treatment. Representative results of triplicate independent experiments. Scale bar = 100 µm. Magnification = ×10.

To evaluate cellular uptake of immunoliposomes by A172 cells, we encapsulated the immunoliposomes with DiOC_18_(3), which allowed fluorescent visualization and quantitative assessment of cellular uptake (Fig. 5a). Remarkably, at 10 µM liposomal (phospholipid) concentration, 214D8-conjugated liposome was gradually taken up by the cells, reaching its apex at 69 h (Fig. 5b). When compared with the control mIgG immunoliposome, 214D8- and 6E1-conjugated liposomes exhibited 2.21- (214D8: 66 h) and 4.25-fold (6E1: 51 h) increases of the cellular uptake (Fig. 5c). Phase contrast and fluorescent images at 72 h after treatment are shown in Fig. 5d, indicating enhanced cellular uptake of fluorescence-labeled immunoliposome. With increase of liposomal concentration to 30 µM, the control mIgG-conjugated liposome was also taken up by the cells, minimizing the ratio between 214D8- or 6E1-conjugated liposomes and the control. Since the immunoliposomes were permanently administrated without washing steps, these results indicated that lowest concentration tested was sufficient to validate functionality of antigen–antibody dependent cellular uptake of the immunoliposome.

### Anti-CD71 antibody-conjugated liposome encapsulating doxorubicin demonstrated enhanced cytotoxicity

**Figure 6:**
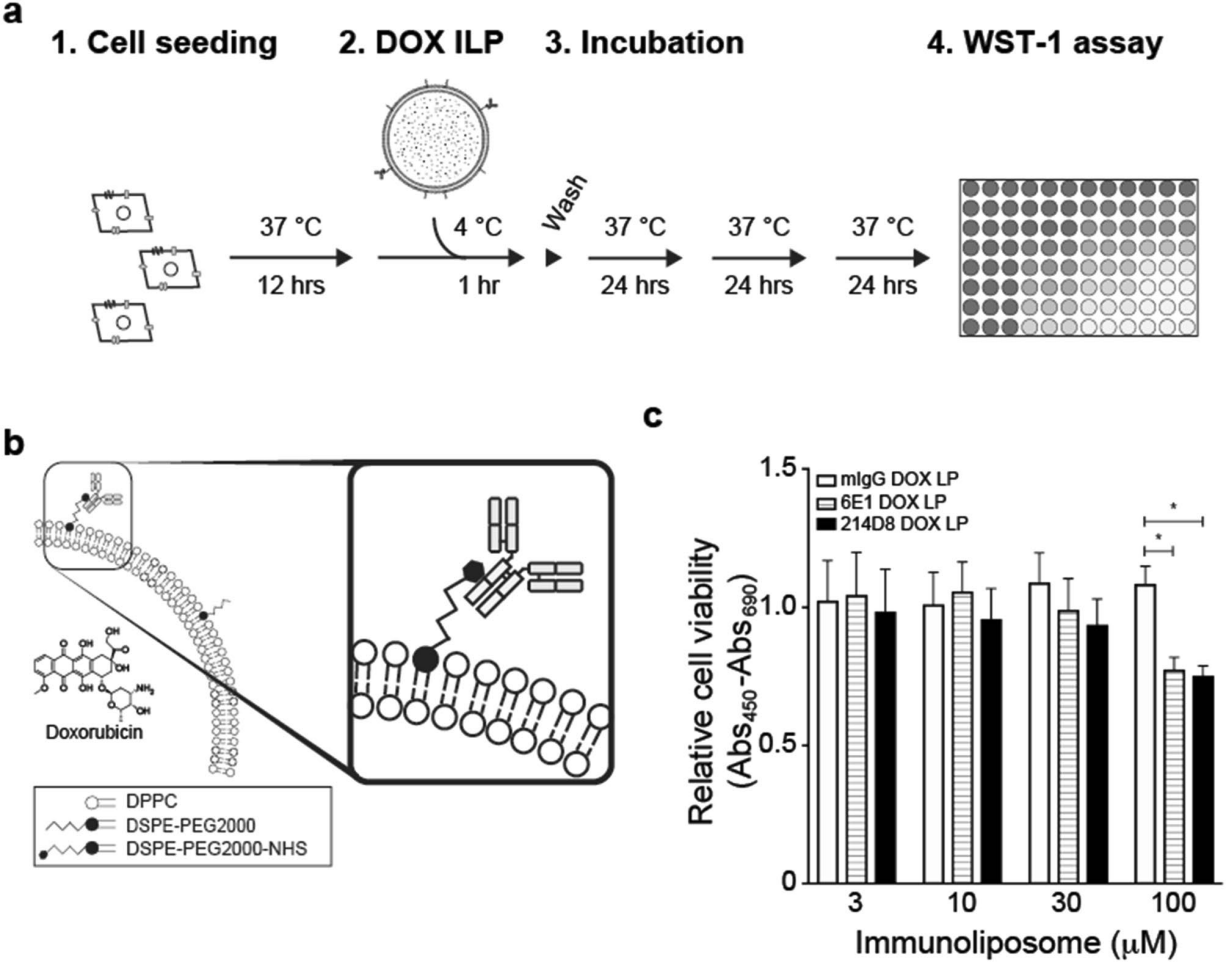
Enhanced cytotoxicity by doxorubicin-encapsulated 214D8-conjugated liposome. **(a)** Schematic diagram of cytotoxicity analysis. A172 cells were treated with 3 to 100 µM immunoliposomes encapsulating doxorubicin for 1 h, washed with culture media, incubated at 37 °C for 3 days, and analyzed by WST-1 assay. **(b)** Schematic diagram of doxorubicin-encapsulated immunoliposomes. Quantitative analysis of the immunoliposomes indicated that approximately 2000 doxorubicin molecules were encapsulated per antibody. **(c)** Reduced relative cell viability by 214D8-conjugated liposome. Treatment with 100 µM 214D8- or 6E1-conjugated liposomes (n = 5 per administration) resulted in 31% and 29% reduction of relative cell viability. Representative results of duplicate independent experiments. Data represent AVG ± SD.

Since enhanced cellular uptake of the immunoliposomes was demonstrated, we next sought to determine their capacities to deliver a therapeutic drug into the cells. A172 cells were seeded overnight, treated with 3 to 100 µM immunoliposomes encapsulating doxorubicin at 4 °C for 1 h, washed with culture media once, and incubated at 37 °C for 3 days (Fig. 6a,b). As shown in Fig. 6c, at 100 µM, 214D8- and 6E1-conjugated liposomes demonstrated 31% and 29% reduced levels of relative cell viability. Lower concentrations of the immunoliposomes did not affect the relative cell viability. Quantitative analyses of encapsulated doxorubicin and conjugated antibody indicated 119 µg doxorubicin/16 µg antibody/1 µmol phospholipid for 214D8-conjugated liposome, 127 µg doxorubicin/18 µg antibody/1 µmol phospholipid for 6E1-conjugated liposome, and 128 µg doxorubicin/17 µg antibody/1 µmol phospholipid for mIgG-conjugated liposome. Encapsulation efficiencies of doxorubicin were 44%, 47%, and 47%, respectively.

## Discussion

Through generation of anti-U87 hybridoma library and screening of hybridoma supernatants treated with DT3C, we identified functional 214D8 antibody that targeted the surface molecule of U87 cells and were internalized to induce cytotoxicity. These findings provided evidence, such as targeting of cell surface molecules and cellular internalization, supporting 214D8 as a potential drug delivery agent. Subsequent biochemical and cellular analyses strongly indicated that the antigen of 214D8 antibody was CD71/TFRC. CD71 is a type 2 membrane protein expressed as a homodimer in the cell membrane that mediates internalization of diferric holo-transferrin by clathrin-mediated endocytosis^11,12^. While generally ubiquitous, CD71 is expressed on the surface of immature erythroid cells and placental tissues of healthy individuals^13,14^. Additionally, CD71 is expressed on the surface of brain capillary endothelial cells that form the blood–brain barrier (BBB), as well as choroid plexus epithelial cells that form the blood–cerebrospinal fluid (CSF) barrier; both of which play an important role in the developing brain^15^.

A majority of research efforts on CD71 has been conducted in the cancer research because increased expression levels of CD71 have been reported in the multiple forms of cancer including glioblastoma^16,17^. Since expression of CD71 has been demonstrated in the human glioblastoma cell line U87^18^, and we used U87 for immunization of BALB/c mice, it is conceivable that we obtained the monoclonal antibody against CD71 through our screening strategy. Given the enhanced expression of CD71 in the various types of cancer, CD71 has generated a great interest in producing monoclonal antibodies that can either induce cytotoxicity of cancer cells through direct inhibition of the receptor function or deliver therapeutic agents^19^. In case of 214D8 antibody, while we observed strong immunoreactivities against A172, U87, and SH-SY5Y cells by flow cytometry, we did not detect cytotoxicity of these cells by administration of the antibody alone. These findings suggested that 214D8 is more suited for delivery of therapeutic agents. In fact, when 214D8 was formed with DT3C, the immunotoxin was able to induce cytotoxicity among all four cell lines tested, confirming the delivery and internalization of the immunotoxin.

To maximize drug load while minimizing structural changes and instability of the antibody, we generated a drug delivery modality that consisted of phospholipid bilayer liposome conjugated with monoclonal antibody that was collectively designed for targeted delivery to the cells expressing its corresponding antigen. There are multiple advantages to liposomes including capacity to compartmentalize both hydrophilic and hydrophobic drugs^20–22^. In principle, various types of clinically approved or presently developed therapeutic agents including levodopa are potential drug candidates for liposomal encapsulation^23,24^, as well as contrast agents or radionuclides^25–27^ for diagnostic and theranostic application. Through the ethanol injection method, followed by extrusion and antibody conjugation, we were able to rapidly and efficiently generate immunoliposomes with multiple types of antibodies. When compared amongst mIgG-, 6E1-, and 214D8-conjugated liposomes, physiological properties did not differ significantly. Previously, antibody conjugation ranged from 6 antibodies per a liposome^28^ to approximately 50 antibodies per a liposome^29^. Given the antibody conjugation percentages and the quantity of liposome particles, our optimized method allowed conjugation of approximately 100 antibodies per a liposome.

To test our concept of maintaining functionality of antigen–antibody dependent binding, and maximizing drug load while minimizing structural changes and instability of antibody, we treated A172 cells with DiOC_18_(3)-encapsulated immunoliposomes. Interestingly, we observed antigen–antibody dependent uptake wherein 214D8- and 6E1-conjugated liposomes exhibited 2.21- and 4.25-fold increases of the cellular uptake when compared with the control immunoliposome. We next determined antigen–antibody dependent delivery of a therapeutic drug by encapsulating doxorubicin into our immunoliposomes. Doxorubicin was selected since liposomal formulation of this anti-cancer agent (Doxil) has already been well-characterized and FDA-approved^30–32^. Remarkably, when A172 cells were treated with 100 µM 214D8- or 6E1-conjugated liposomes, we observed 31% and 29% reduction of relative cell viability. Quantitative assessment of the doxorubicin-encapsulated immunoliposomes demonstrated that approximately 2000 doxorubicin molecules were encapsulated per antibody. Taken together, these findings confirm the potential of our immunoliposome as an antigen–antibody dependent drug delivery modality that conceivably can overcome the limitation imposed by DAR.

As one of the potential targets of anti-CD71 antibody, the BBB is a selective barrier between systemic blood circulation and brain parenchyma, and because of the complexity of the central nervous system and the impermeable BBB, delivery of therapeutic drugs across the BBB is a well-documented hurdle^33–35^. Despite the notion that CD71 is expressed on the surface of the BBB, only recently, technological tools have been reconceptualized to exploit the receptor-mediated transcytosis with promising potentials^36^ including favorable features of anti-CD71 antibody variants with low affinity that allowed the increased release from the BBB into the brain^37,38^. Recently, Johnsen et al.^29^ reported that intravenous injection of OX26 (a mouse monoclonal antibody against rat CD71)-conjugated and oxaliplatin-loaded liposome resulted in detection of higher concentration of platinum in the rat brain parenchyma compared to the control rat IgG-conjugated liposome. Additionally, a BBB-penetrating fusion protein, JR-141, which is composed of anti-hCD71 antibody and intact enzyme has recently been reported for treatment of lysosomal storage disease^39^. As a new modality, anti-CD71 targeted immunoliposome in this study would become advantageous format with high capacity for encapsulation.

In summary, through our screening strategy, we obtained functional 214D8 antibody that targeted the cell surface molecule CD71 with capacity for cellular internalization. To overcome the limitation of DAR, we generated anti-CD71 antibody- conjugated liposome, evaluated its cellular internalization property, and observed enhanced cellular uptake and cytotoxicity. Taken together, our pipeline with optimized method is a rapid and efficient procedure to isolate potent antibodies with therapeutic potentials as ADCs and to synthesize functional immunoliposomes.

## Methods

### Cell culture

A172, CHO, SH-SY5Y, H4, and U87 were purchased from the American Type Culture Collection (Rockville, MD, USA). A172 and H4 cells were cultured in DMEM (Nacalai Tesque, Inc.; Kyoto, Japan) supplemented with 10% fetal bovine serum (FBS; Life Technologies Corp.; Grand Island, NY, USA) and a mixture of penicillin, streptomycin, and amphotericin B (PSA; Nacalai Tesque, Inc.; Kyoto, Japan). SH-SY5Y and CHO were cultivated in DMEM/Ham’s F-12 (Nacalai Tesque, Inc.; Kyoto, Japan) supplemented with 10% FBS and PSA. U87 was cultivated in EMEM (FUJIFILM Wako Pure Chemical Corp.; Osaka, Japan) supplemented with 10% FBS, PSA, sodium pyruvate (Nacalai Tesque, Inc.; Kyoto, Japan), and MEM non-essential amino acids (Nacalai Tesque, Inc.; Kyoto, Japan). All cell lines were maintained at 37 °C in 5% CO_2_ incubator (MCO-175; Sanyo Electric Co., Ltd.; Osaka, Japan).

### Production of hybridoma library

BALB/c mice (Japan SLC, Inc.; Hamamatsu, Japan) aged 7–10 weeks were immunized weekly or bi-weekly for at least 19 weeks by intra-peritoneal injection of 5 × 10^6^ U87 cells. These animals received boost immunization at 3 days before being sacrificed by dislocation or injection of pentobarbital (Kyoritsu Seiyaku Corp.; Tokyo, Japan). Immunized 1 × 10^8^ splenocytes and 3 × 10^7^ P3U1 myeloma cells were fused with Hybri-Max PEG/DMSO solution (Sigma-Aldrich; St. Louis, MO, USA). Hybridoma library was cultured in RPMI-1640 media (Nacalai Tesque, Inc.; Kyoto, Japan) supplemented with 10% Hyclone Super Low IgG Defined FBS (GE Healthcare Life Sciences; Utah, USA), PSA, sodium pyruvate, MEM non-essential amino acids, 2-mercaptoethanol (Life Technologies Corp.; Grand Island, NY, USA), and hypoxanthine-aminopterin-thymidine (HAT) (Life Technologies Corp.; Grand Island, NY, USA), and incubated at 37 °C in the 5% CO_2_ incubator for approximately a week for proper HAT selection. The library was screened by using toxin DT3C as described below. For cloning, positive hybridomas from the library were subjected to limiting dilution. Immunoglobulin (Ig) subclass was determined by using IsoStrip mouse monoclonal antibody isotyping kit (Sigma-Aldrich; St. Louis, MO, USA). The animal experimental protocol used in this study was approved and carried out in accordance with the guidelines and regulations from the institutional review board for animal experiment at Juntendo University School of Medicine (#1294), as well as the ARRIVE guidelines.

### Immunotoxin assay

Recombinant DT3C was purified as described previously^10^. Supernatants from the hybridoma library or purified antibodies were pre-incubated with DT3C at 37 °C for 30 min in the CO_2_ incubator. Antibody:DT3C immunocomplexes and 1.0 × 10^4^ cells were seeded simultaneously in the 96-well plates. After 3 days of cultivation in the CO_2_ incubator, cell viability was measured by using WST-1 reagent [5 mM WST-1 (Dojindo Laboratories; Kumamoto, Japan), 2 mM 1-methoxy PMS (Dojindo Laboratories; Kumamoto, Japan), 18 mM HEPES (pH 7.4)]. Absorbance at 450 nm and 690 nm (as reference) was measured by using SpectraMax iD3 plate reader (Molecular Devices; San Jose, CA, USA). Relative cell viability was calculated, and data analysis was performed by using Prism 7 ver. 7.0 (GraphPad Software; San Diego, CA, USA; https://www.graphpad.com/).

### Purification of antibody

Monoclonal antibody was purified as described previously^6^. Briefly, hybridoma supernatant was diluted at the ratio of 1:1 with protein G IgG binding buffer (0.1 M sodium acetate buffer, pH 5.2), and incubated with COSMOGEL Ig-Accept protein G beads (Nacalai Tesque, Inc.; Kyoto, Japan) at 4 °C for 12 h. The bound antibody was eluted by using protein G IgG elution buffer (Thermo Fisher Scientific; Waltham, MA, USA), and rapidly neutralized with 1 M Tris–HCl (pH 9.0). Fractions containing 214D8 antibody were filtered, concentrated, and sterilized through 0.45-μm cellulose acetate filters (DISMIC-03CP; Advantec, Tokyo, Japan). Antibody concentration was measured by using NanoDrop 1000 (Thermo Fisher Scientific; Waltham, MA, USA).

### Biotinylation of cell surface proteins

Biotinylation of cell surface proteins was performed as described previously^6^. Briefly, approximately 5 × 10^6^ A172, CHO mock or CHO hCD71-OFP cells were incubated with 5 ml PBS (−) containing 1.0 mg EZ-Link sulfo-NHS-biotin (Thermo Fisher Scientific; Waltham, MA, USA) at room temperature for 30 min on the horizontal shaker (NJ-022NS; Nisshin Rika; Tokyo, Japan). The biotinylated cells were then treated with PBS (−) containing 100 mM glycine, and lysed in the lysis buffer [50 mM Tris–HCl (pH 7.6), 150 mM NaCl, 1% NP40, and protease inhibitor cocktail (Nacalai Tesque, Inc.; Kyoto, Japan)]. After incubation at 4 °C for 30 min, the cell lysate was centrifuged at 20,400×*g* for 20 min to obtain NP40-soluble fraction.

### Immunoprecipitation and immunoblotting

The NP40-soluble lysate or purified recombinant hCD71 (rhCD71; 77.4 kDa; Sino Biological Inc.; Peking, China) diluted in the lysis buffer was incubated with Ig-Accept protein G beads at 4 °C for 30 min to minimize non-specific binding. Subsequently, proteins in the lysate was immunoprecipitated with control mouse IgG (mIgG; Southern Biotech; Birmingham, AL, USA), 6E1 (prepared in-house) or 214D8 antibodies, followed by binding of these antibodies with COSMOGEL Ig-Accept protein G beads. After the binding step, the beads were washed with 1% NP40 lysis buffer without protease inhibitor, and heat-denatured with appropriate volume of sample buffer solution with reducing reagent (6x) (Nacalai Tesque, Inc.; Kyoto, Japan). SDS polyacrylamide gel electrophoresis (PAGE) was performed by using a 5–20% gradient gel (Nacalai Tesque, Inc.; Kyoto, Japan). Proteins were transferred to Immobilon-P PVDF membrane (Millipore; Bedford, MA, USA) with semi-dry transfer buffer [192 mM glycine, 25 mM Tris–HCl (pH 7.4)], and treated with 5% skim milk for blocking. For detection of hCD71 in the NP40-soluble lysate, the protein was probed with streptavidin-HRP conjugate (GE Healthcare Japan, Ltd.; Tokyo, Japan) at room temperature for 1 h, and detected by using chemiluminescent reagent. For detection of purified rhCD71, the protein was probed with rabbit anti-human TFRC polyclonal antibody at 4 °C overnight (1:5000; Sino Biological Inc.; Peking, China), followed by donkey anti-rabbit IgG HRP (1:100,000; Jackson ImmunoResearch Laboratories, Inc.; West Grove, PA, USA) at room temperature for 1 h, and detected by using chemiluminescent reagent. Chemiluminescent images were captured with biomolecular imager (ImageQuant LAS4000; GE Healthcare Japan, Ltd.; Tokyo, Japan).

### Sandwich ELISA

To coat a polystyrene high bind 96-well microplate with capture antibody, the rabbit anti-human TFRC polyclonal antibody (1:1000) was added to the wells and incubated at 4 °C overnight. The plate was then blocked with 5% skim milk at room temperature for 1 h. The purified rhCD71 was serially diluted in PBS (pH 7.4) containing 2% skim milk, transferred to the microplate wells, and incubated at 37 °C for 1.5 h. Subsequently, the purified rhCD71 was detected by control mIgG, 6E1 or 214D8 antibodies (600 ng/well) at room temperature for 2 h, and probed with goat anti-mouse IgG HRP (1:5000; Jackson ImmunoResearch Laboratories, Inc.; West Grove, PA, USA) at room temperature for 1 h. After washing, the microplate was incubated with TMB (1-component) peroxidase substrate solution (KPL, Inc.; Gaithersburg, MD, USA). Absorbance at 450 nm after adding 0.12 N HCl as stopping solution was measured by using SpectraMax ABS Plus plate reader (Molecular Devices; San Jose, CA, USA).

### Flow cytometry

For flow cytometry analysis, 2.5 × 10^5^ cells were stained with 2.5 µg of mIgG, isotype control mIgG1 (Clone MOPC-21; Biolegend; San Diego, CA, USA), 6E1 or 214D8 primary antibodies. FITC-conjugated rabbit anti-mouse polyclonal IgG (H + L chain) antibody (Medical & Biological Laboratories Co., Ltd.; Nagoya, Japan) was employed as secondary antibody. In case of DiOC_18_(3)-labeled immunoliposomes, 2.5 × 10^5^ cells were stained with immunoliposomes containing 0.25 µg, 0.83 µg or 2.5 µg of antibodies. Live cells were dissociated with TripLE reagent (Invitrogen; Carlsbad, CA, USA), stained with primary and secondary antibodies for 30 min each on ice, washed with PBS (−), and measured by using FACSCalibur flow cytometer (BD Biosciences; San Diego, CA, USA). Data analysis was performed by using FlowJo software ver. 10.3.0 (FlowJo, LLC; Ashland, OR, USA; https://www.flowjo.com/).

### Transfection of CHO cell line

To confirm immunoreactivity of 214D8 antibody, 1 × 10^5^ CHO cells were transfected with 3 µg of expression plasmid (Sino Biological Inc.; Beijing, China) encoding human transferrin receptor fused to orange fluorescent protein (OFP) or pcDNA3 as a control (Invitrogen; Carlsbad, CA, USA) by using Polyethylenimine Max (Polysciences, Inc.; Warrington, PA, USA). The cells were analyzed by flow cytometry at 3 days after transfection.

### Immunocytochemistry

A172 cells (1 × 10^5^ cells) were transferred onto pre-washed 0.13–0.17 mm-thick coverslips (1-S; Matsunami; Osaka, Japan), and incubated at 37 °C overnight. After fixation with 4% PFA, the coverslips were incubated with blocking solution [0.2% gelatin, 1% BSA, 0.05% Tween-20 in PBS (−)], and treated with 1 μg primary antibodies at 4 °C overnight. The coverslips were then incubated with 1 μg Alexa555-conjugated donkey anti-mouse IgG (H + L) secondary antibody (Invitrogen; Carlsbad, CA, USA) at room temperature for 1 h, and mounted with VECTASHIELD HardSet Antifade Mounting Medium with DAPI (Vector Laboratories, Inc; Burlingame, CA, USA). Multi-color images were obtained by using Zeiss Axio Imager 2 fluorescent microscope (Carl Zeiss Microscopy GmbH; Jena, Germany) equipped with AxioCam MRc digital camera (Carl Zeiss Microscopy GmbH; Jena, Germany) and 40 × objective lens.

### Preparation of DiOC_18_(3)-encapsulated liposome

1,2-Dipalmitoyl-sn-glycero-3-phosphocholine (DPPC) (Yuka Sangyo Co., Ltd.; Tokyo, Japan), cholesterol (Sigma-Aldrich; St. Louis, MO, USA), DSPE-PEG2000 (Yuka Sangyo Co., Ltd.; Tokyo, Japan), and DSPE-PEG2000-NHS (Yuka Sangyo Co., Ltd.; Tokyo, Japan) at molar ratio of 60:35:4:1 were dissolved in ethanol. To 25 µmol total lipids, 0.48 mg DiOC_18_(3) (Life Technologies Corp.; Eugene, OR, USA) was added. The lipid + DiOC_18_(3) ethanol mixture was rapidly injected into the solution containing 50 mM HEPES buffer (pH 7.4) with 5% mannitol or 250 mM ammonium sulfate buffer (pH 5.5) with 5% mannitol at 55 °C. The resulting lipid suspension was passed through LIPEX extruder (Northern Lipids, Inc.; Burnaby, Canada) through a series of 0.05 µm, 0.1 µm, and 0.2 µm Whatman Nuclepore polycarbonate filters (GE Healthcare Japan, Ltd.; Tokyo, Japan) for 5 times at 45 °C.

### Conjugation of antibodies to DiOC_18_(3)-encapsulated liposome

Immediately following the extrusion, the liposome solution was mixed with 9% NaCl solution to make final concentration of 0.9% NaCl, and ultracentrifuged at 100,000×*g* for 20 min. The pelleted liposomes containing 0.25 µmol DSPE-PEG2000-NHS were resuspended with PBS (−), mixed with 2.5 nmol antibody, incubated at room temperature for 1 h, and further incubated at 4 °C for 12 h. The liposome + antibody solution was mixed with 9% NaCl solution to make final concentration of 0.9% NaCl, and ultracentrifuged (Himac CS 150GXL Micro Ultracentrifuge; Hitachi Koki Co., Ltd.; Tokyo, Japan) at 100,000×*g* for 20 min to pellet liposome, and remove free DiOC_18_(3) and antibody. The resulting immunoliposomes were stored in PBS (−) at 4 °C until further use. Phospholipid concentration was measured by LabAssay Phospholipid kit (Wako Pure Chemical Industries, Ltd.; Osaka, Japan), and protein concentration was determined by Protein Assay BCA Kit (Nacalai Tesque, Inc.; Kyoto, Japan). Mean diameter and number of the liposomes were estimated by using Nanosight LM10 nanoparticle analysis system with NTA 1.5 analytical software ver. 3.2 (Nanosight Ltd.; Salisbury, UK; https://www.malvernpanalytical.com/en). Zeta potential was measured by using Zetasizer Nano (Malvern Instruments Ltd.; Worcestershire, UK).

### Active loading of doxorubicin into immunoliposome

Following the conjugation of antibodies to liposomes as described above, the immunoliposomes were ultracentrifuged at 100,000×*g* for 20 min, and the pelleted immunoliposomes were resuspended with 10 mM histidine (pH 6.5) + 10% sucrose solution. The immunoliposomes were mixed with doxorubicin hydrochloride (Toronto Research Chemicals, Inc.; Toronto, Canada) at the concentration of 0.2 mg doxorubicin/1 mg phospholipid^40^, and incubated at 42 °C overnight. The resulting doxorubicin-encapsulated immunoliposomes were ultracentrifuged at 100,000×*g* for 20 min three times, and stored in 10 mM histidine (pH 6.5) + 10% sucrose solution at 4 °C until further use. To quantify doxorubicin, the immunoliposomes were treated with 2% Tween-20 solution, and incubated at 60 °C for 30 min. After disruption of liposomal lipid bilayers, absorbance at 495 nm corresponding to doxorubicin was measured.

### Analysis of equilibrium dissociation constant

To determine equilibrium dissociation constant of immunoliposomes, 2 × 10^6^ A172 cells were transferred to a tilted 100-mm cell culture dish, and incubated at 37 °C overnight. The seeded dish was placed into LigandTracer (Ridgeview Instruments AB; Vange, Sweden), and baseline, association phase, and dissociation phase were determined for at least 1 h. For association phase, 30 µM, 100 µM, and 300 µM immunoliposomes were applied. Association rate constant (K_a_), dissociation rate constant (K_d_), and equilibrium dissociation constant (K_D_) were analyzed by using TraceDrawer software ver. 1.6 (Ridgeview Instruments AB; Vange, Sweden; https://www.ligandtracer.com/).

### Cellular uptake of DiOC_18_(3)-encapsulated immunoliposomes

For cellular uptake analysis, immunoliposomes at the concentration of 1–30 µM phospholipids and 1.0 × 10^4^ A172 cells were seeded simultaneously in the 96-well plates, and transferred into IncuCyte ZOOM (Essen BioScience, Inc.; Ann Arbor, MI, USA). After 3 days of cultivation, green fluorescent areas were measured as green object confluency (GOC), and data analysis was conducted by using IncuCyte ZOOM software ver. 2018A (Essen BioScience, Inc.; Ann Arbor, MI, USA; https://www.essenbioscience.com/en/).

### Cytotoxicity of doxorubicin-encapsulated immunoliposomes

For cytotoxicity analysis, 1.0 × 10^4^ A172 cells were seeded in the 96-well plates and incubated at 37 °C overnight, followed by the treatment with doxorubicin-encapsulated immunoliposomes at the concentration of 3–100 µM phospholipids at 4 °C for 1 h. The plates were then washed once with culture media, and incubated at 37 °C for 3 days. After 3 days of cultivation, relative cell viability was measured by using WST-1 reagent, and data analysis was performed by using Prism 7.

### Statistical analysis

For cytotoxicity analysis of doxorubicin-encapsulated immunoliposomes, two-way ANOVA with Sidak test was performed for multiple group comparisons. *P* < 0.01 was determined as statistically significant.

**Table 1:** Characterization of immunoliposomes.

	No conjugation	mIgG LP	6E1 LP	214D8 LP
Diameter (nm)	99.9 ± 3.2	136.8 ± 1.2	139.4 ± 2.2	127.1 ± 20.6
SD (nm)	27.1 ± 0.4	34.4 ± 1.5	35.1 ± 3.1	55.9 ± 2.7
Number of particles (count/µl)	6.95e^9^	9.90e^9^	6.25e^9^	1.02e^9^
Zeta potential (mV)	− 41.1 ± 0.2	− 30.4 ± 0.7	− 28.5 ± 0.8	− 32.0 ± 1.0
Proteins/phospholipids (µg/µmol)	n/a	19.1 ± 3.7	17.2 ± 2.2	14.9 ± 3.1
Antibody conjugation (%)	n/a	76.3 ± 14.3	68.8 ± 8.8	59.8 ± 12.2
Number of antibodies (antibodies/liposome)	n/a	77.4	110.5	589.2

## Supplementary Information

Supplementary Figures.
