# Supplementary material for: Novel method for screening functional antibody with comprehensive analysis of its immunoliposome
Source: Sci Rep. 2021 Feb 25;11:4625. doi: 10.1038/s41598-021-84043-w (PMC7907096; doi:10.1038/s41598-021-84043-w)
Supplement: Supplementary file 1 — Supplementary Figures. [file 41598_2021_84043_MOESM1_ESM.docx]

**Novel method for screening functional antibody with comprehensive analysis of its immunoliposome**

Shusei Hamamichi^1^, Takeshi Fukuhara^2,3,*^, Izumi O. Umeda^4,5^, Hirofumi Fujii^5^, Nobutaka Hattori^2,3^

^1^ Research Institute for Diseases of Old Age, Juntendo University School of Medicine, Tokyo 113-8421 Japan

^2^ Department of Neurology, Juntendo University School of Medicine, Tokyo 113-8421 Japan

^3^ Department of Research for Parkinson's Disease, Juntendo University Graduate School of Medicine, Tokyo 113-8421, Japan

^4^ Kavli Institute for the Physics and Mathematics of the Universe, The University of Tokyo, Kashiwa, Chiba 277-8583 Japan

^5^ Division of Functional Imaging, Exploratory Oncology Research and Clinical Trial Center, National Cancer Center, Kashiwa, Chiba 277-8577 Japan

**Corresponding author:**

Takeshi Fukuhara, Ph.D

Department of Neurology, Juntendo University Graduate School of Medicine

2-1-1 Hongo, Bunkyo-ku, Tokyo 113-8421, Japan

Phone: +81-3-5802-2731; Fax: +81-3-5800-0547

E-mail: noantibody-noscience@umin.ac.jp


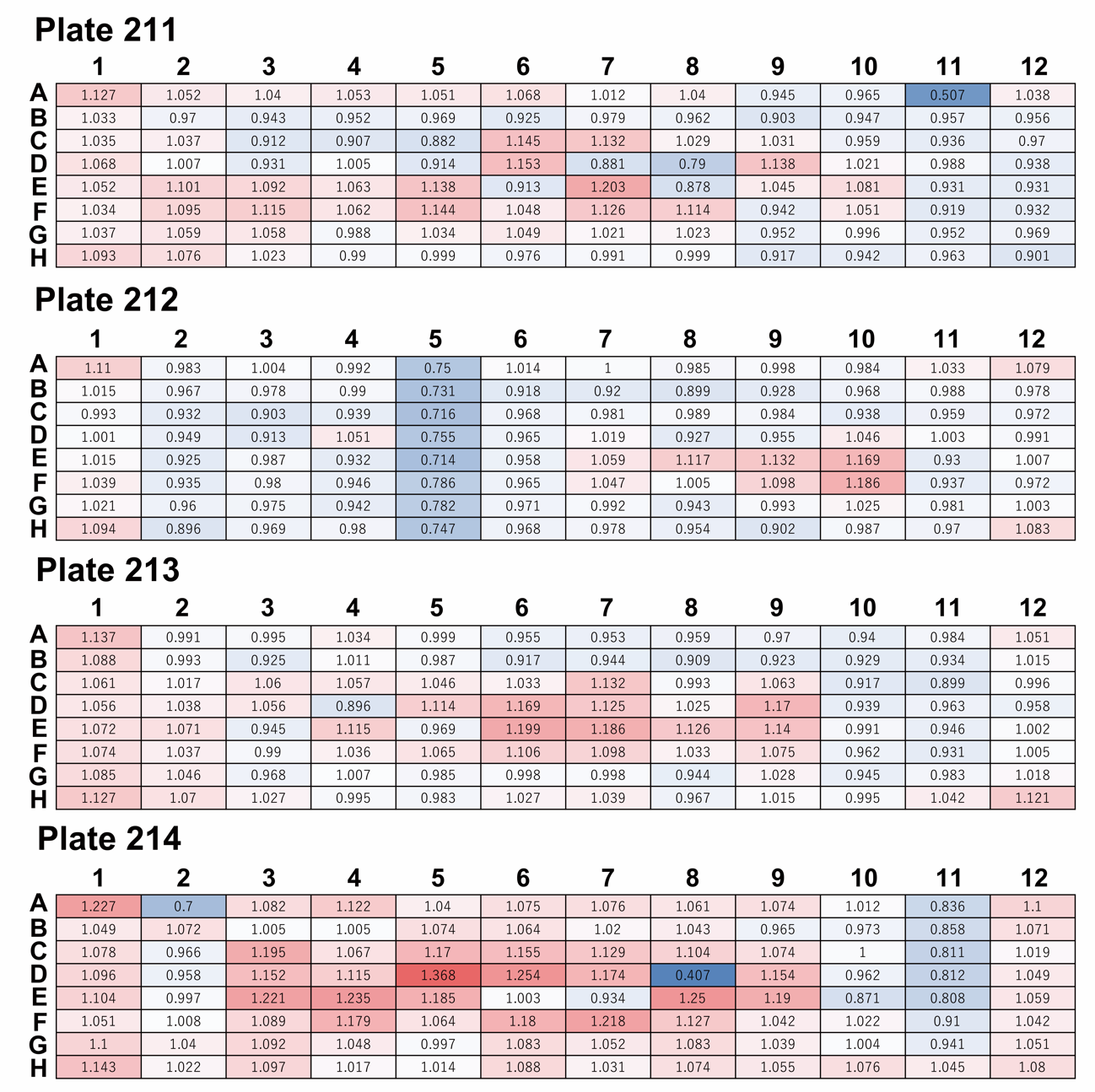


**Supplementary Figure 1S. Representative data of primary immunotoxin screening.** Immunotoxin screening of U87 cells by using immunotoxins formed between supernatants from 4 96-well plates and DT3C revealed 214A2 and 214D8 as putative hybridomas that secreted functional antibodies with DT3C-dependent cytotoxicity. As a positive control, 6E1 (100 ng/well) was placed in 211A11, and as a negative control mIgG (100 ng/well) was placed in 211A12.

**Supplementary Figure 2S. Representative images of immunotoxin assay.** **(a)** Phase contrast images of A172, U87, SH-SY5Y and H4 cell lines after antibody treatment. Administration of antibodies at 300 ng/well did not induce cytotoxicity (n = 3 per administration). Representative results of duplicate independent experiments. Scale bar = 100 μm. **(b)** Phase contrast images of A172, U87, SH-SY5Y and H4 cell lines after antibody:DT3C treatment. Through formation of immunotoxins at 300 ng/well, both 214D8:DT3C and 6E1:DT3C induced cytotoxicity in all four cells lines tested (n = 3 per administration). As a negative control, mIgG:DT3C did not induce cytotoxicity. Representative results of duplicate independent experiments. Scale bar = 100 μm. Magnification = 4x.

**Supplementary Figure 3S. Comparison of median fluorescent intensity among immunoliposomes.**  **(a)** A172 cells were tested by staining with control mIgG-conjugated (dotted white), 6E1-conjugated (white) or 214D8-conjugated liposomes (gray). Data of FITC fluorescent intensity (FL1) were indicated as histograms. Representative results of duplicate independent experiments. **(b)** Median fluorescent intensity of immunoliposomes and liposome without antibody conjugation. Concentrations of immunoliposomes based on antibody (Ab) concentrations (μg) are represented in x-axis. Representative results of duplicate independent experiments. **(c)** Median fluorescent intensity of immunoliposomes and liposome without antibody conjugation. Concentrations of immunoliposomes based on phospholipid (PL) concentrations (μmol) are represented in x-axis. Representative results of duplicate independent experiments.

**Figure 2b**





**Figure 2c**





**Supplementary Figure 4S. Original unprocessed full immunoblot images of the main manuscript.** Dotted areas are represented in the main manuscript.
